# Evaluation of Three Different Selective Media for Enumeration of *Clostridium perfringens* in Untreated and Treated Wastewater

**DOI:** 10.3390/pathogens13070526

**Published:** 2024-06-21

**Authors:** A. J. Karon, Emily S. Bailey, Mark D. Sobsey

**Affiliations:** 1 Department of Environmental Sciences and Engineering, Gillings School of Global Public Health, The University of North Carolina at Chapel Hill, Chapel Hill, NC 27599, USA; 2 Department of Public Health, College of Pharmacy & Health Sciences, Campbell University, Buies Creek, NC 27506, USA

**Keywords:** *C. perfringens*, indicators, protozoan parasite, monitoring

## Abstract

Current and emerging legislation in North Carolina and other regions calls for the enumeration of *Clostridium perfringens* as a surrogate indicator for protozoan parasites in various types of waters. Past studies that have evaluated selective media for the detection of this bacterium have provided limited, conflicting, and inconclusive results. In this study, membrane filtration was used to enumerate *C. perfringens* as culturable spores or total culturable cells in 19 samples of untreated and 25 samples of partially treated wastewaters on 3 candidate media, Tryptose Sulfite Cycloserine Agar (TSC), CP ChromoSelect Agar (CPCS), and membrane *Clostridium perfringens* Agar (m-CP) in parallel, and the results were compared. Presumptive isolates from each agar were further subjected to phenotypic confirmation tests for acid phosphatase production and stormy fermentation to further determine the performance of each agar. The CPCS agar was determined to have the highest enumerative capacity of total *C. perfringens* cells when compared to both TSC agar and m-CP agar (*p*-value < 0.05), but there was no significant difference in its ability to detect spores when compared to TSC agar (*p*-value > 0.05). The overall specificity of CPCS agar as determined by agreement of results from both confirmation tests was 0.81, while the specificity of TSC agar was only 0.28. Based on its performance, ease of preparation and use and consistency of colony characteristics, CPCS agar is recommended as the preferred medium for *C. perfringens* enumeration in wastewater.

## 1. Introduction

As water scarcity becomes a more prevalent global issue in the face of climate change and water source depletion, the need for alternative sources of water for drinking, industrial purposes, and agriculture is increasingly important. To address this need, treated wastewater is often suggested as an alternative source for augmenting drinking water supplies and other beneficial uses. In 2011, North Carolina passed legislation revising the regulation of reclaimed water that specified a new, higher quality reclaimed water with expanded allowable uses of such reclaimed water for agricultural and industrial purposes [1]. Following this action, in 2014, the North Carolina legislature again expanded the allowable uses for this reclaimed water to include mixing with source waters for potable drinking water supplies at an approved ratio to then be further treated to produce drinking water [2]. Combined, these two actions allow for potable reuse of reclaimed water in North Carolina.

The North Carolina-sponsored legislation enabling expansion of allowable uses for reclaimed water also established specific quality guidelines for the higher quality reclaimed water, named type 2 reclaimed water, as the only category of reclaimed water that would be allowed for these expanded uses [1]. In order to meet the criteria for type 2 reclaimed water, these waters had to be treated by tertiary processes and dual disinfection (i.e., chlorine disinfection and ultraviolet (UV) disinfection or allowable substitutes) and have more extensive quality testing than previously necessary for other treated wastewaters. Included among these new quality tests was testing the type 2 tertiary treated wastewater for *Clostridium perfringens*, a spore-forming bacterium that serves as a surrogate indicator for protozoan parasite pathogens. For water to be considered type 2, reclaimed water treatment had to demonstrate a greater than 4 log_10_ reduction in *C. perfringens* from raw sewage and produce water with a geometric mean of no more than 5 CFU/100 mL with a daily maximum of 25 CFU/100 mL of treated water [1].

While these new regulations contained specifications regarding the ultimate quality of the water to satisfy the type 2 quality requirements, there was no mention of the appropriate methods used for proper enumeration of *C. perfringens* in reclaimed water and wastewater samples. Furthermore, US EPA methods and the Standard Methods for the Examination of Water and Wastewater (SMEWW) do not provide or cite official documentation for the recommended methods to enumerate *C. perfringens* in treated wastewater samples such as those that will be produced in North Carolina in the coming years. Several different culture methods have been described by US EPA [3], the United Kingdom (the Environment Agency) [4], the International Standards Organization, and Standard Methods for the Examination of Water and Wastewater for *C. perfringens* quantification in other types of waters such as surface waters and drinking waters, but the performance of these methods has not been evaluated for this specific application to reclaimed water. Additionally, new methods for enumeration of *C. perfringens* in water samples continue to be developed, but there has been limited documentation comparing the performance of the different methods available for the detection of this pathogen [5,6,7]. This can cause confusion and uncertainty about how to effectively detect and quantify *C. perfringens* in both treated wastewaters and other types of waters. The purpose of this research was to measure and compare the effectiveness of three different available selective agars for enumeration of *C. perfringens* using membrane filtration and differential/selective culture media in order to determine the best methods for quantifying this bacterium in wastewater and highly treated wastewater samples. These agars were then further validated by subjecting presumptive *C. perfringens* colony isolates from them to several different biochemical confirmation tests to provide a more rigorous assessment of the performance of the selective agars.

## 2. Materials and Methods

### 2.1. Sample Collection

Treated and untreated wastewater effluents were collected at five wastewater treatment/water reclamation plants located in central North Carolina. These facilities were the following: (A) the Orange Water and Sewer Authority wastewater treatment plant (WWTP) in Chapel Hill, (B) the Raleigh Neuse River WWTP, (C) the North Durham Water Reclamation Facility, (D) the Holly Springs WWTP and (E) the North Cary Water Reclamation Facility. 

Initially, seven secondary treated sewage effluent samples were collected from two wastewater treatment plants (A and B) before sand filtration and analyzed for *Clostridium perfringens*. These samples were collected between May and July of 2013. Many of the final tertiary treated and dual disinfected effluent samples had no detectable *C. perfringens* in 100 mL sample volumes, and therefore were below the detection limits of the methods of analysis. Because the determination of the best methods for quantifying *C. perfringens* requires reliable statistical analysis of data on quantifiable levels of the target microorganisms, samples containing sufficient numbers of these target microbes are required. Consequently, microbiological analysis was performed on treated samples collected from earlier stages in the water reclamation process. It was found that clarified secondary effluent collected prior to sand filtration and disinfection consistently yielded sufficient but not excessive concentrations of *Clostridium perfringens*. These same five treatment plant sample locations yielded sufficient concentrations of *Clostridium perfringens* for collected samples that were partially treated as indicated above.

In the second stage of testing, in addition to the samples collected earlier in the treatment system, raw sewage and final reclaimed water samples were also collected for analysis and are included in this report. These samples were collected between August 2013 and August 2014. During this time, 20 secondary treated effluent samples, 5 untreated sewage samples, and 10 final treated reclaimed water samples were analyzed. A third round of sampling for only reclaimed water and untreated sewage took place between February 2015 and July 2015 in which 14 samples of both types were analyzed. Overall, 19 untreated sewage samples, 25 secondary treated effluent samples, and 25 tertiary treated, dual disinfected reclaimed water samples were analyzed (Supplemental Table S1). It is important to note that not all of the reclaimed water samples were treated by identical physical, chemical and biological processes. This table does not include information on steps prior to filtration and disinfection, as these steps were similar at all wastewater treatment plants and included primary clarification (sedimentation), anaerobic digestion of separated wastewater solids, aerobic biological treatment of primary effluent by some form of activated sludge treatment process and secondary clarification after aerobic biological treatment. Each plant, except for plant C, used both free chlorine and UV disinfection in the production of reclaimed water. Only a single disinfection treatment by UV radiation was used at plant C.

### 2.2. Sample Handling

Treated and raw wastewater grab samples were collected from the indicated WWTP sampling points in sterile polypropylene bottles and kept chilled in coolers with ice during transport to our Chapel Hill laboratory. Sampling points were the same as those used by the treatment plants to allow for the utilities’ own analysis of water samples during the various stages of the overall treatment process. The samples were stored at 4 °C upon arrival at the laboratory. *Clostridium perfringens* assays were performed on the day of or the day following sample collection. Samples were collected and analyzed between May 2013 and April 2015.

### 2.3. Membrane Filtration Method

*C. perfringens* spores and total *C. perfringens* (spores plus vegetative cells) were detected and quantified in reclaimed waters by standard membrane filter (MF) methods. These methods were originally developed for the United States Environmental Protection Agency (US EPA) [3] and later modified [8,9] by changes in the composition of the bacteriological medium, m-CP agar. Based on more recent evidence of the inferior performance of the MF method when using m-CP medium, two alternative *C. perfringens* MF media, TSB and CP ChromoSelect agar, were evaluated in parallel with modified m-CP [10,11]. All three agar media were applied simultaneously for MF analysis of samples of reclaimed water and other treated and untreated wastewaters. These analyses focused on samples having *C. perfringens* colony forming unit (CFU) concentrations in the range of the treated effluent limits of 5 (as geometric mean) and 25 (as single sample maximum) per 100 mL as well as at higher concentrations, to facilitate comparisons of agar media performance by statistical analyses. In the MF method, a volume of sample is vacuum filtered through a standard 47 mm diameter, 0.45 μM pore size cellulose ester membrane filter. The membrane filter is placed on the surface of an agar medium for *C. perfringens* (modified m-CP, TSC or CP ChromoSelect) in a Petri dish and the dish is then incubated under anaerobic conditions at 44 °C. *C. perfringens* and related sulphite-reducing clostridia produce characteristic colonies that are then counted.

### 2.4. C. perfringens Analysis Procedures

Procedures for *C. perfringens* detection and enumeration were based on standard membrane filter (MF) methods and then culture on differential/selective media [3,8,9]. The three agar media used for this investigation were Membrane *Clostridium perfringens* (m-CP) agar (Oxoid Microbiology Products, ThermoFisher Scientific, Waltham, MA, USA), CP ChromoSelect Agar (CPCS; Fluka Analytical, ThermoFisher Scientific, Waltham, MA, USA), and Tryptose Sulfite Cycloserine (TSC) agar (EMD Millipore Sigma, Burlington, MA, USA). Agar media were prepared as described by the manufacturer’s instructions, each set of samples was processed with a positive and negative control. Following each assay, other confirmatory analyses were performed to identify false positive and false negative presumptive *C. perfringens* colonies obtained by initial membrane filter analysis and agar media culture. As many as five individual presumptive positive and presumptive negative *C. perfringens* colonies from each of the three *C. perfringens* agar media tested were selected from a given sample and purified by re-streaking onto non-selective Columbia agar (Neogen, Lansing, MI, USA) medium to obtain isolated colonies. Pasteurization of samples, to differentiate spores from vegetative cells, was performed by heating samples at 65 °C for 15 min. 

### 2.5. Confirmation Methods

#### 2.5.1. Acid Phosphatase (AP) Confirmation Method

Upon counting colonies of plates for the three test agars, presumptive positive and negative isolated colonies were then subjected to a confirmation test with Acid Phosphatase (AP) Reagent, based on previously validated methods [12,13]. Presumptive *C. perfringens* colonies from membrane filters were streaked initially onto separate non-selective Columbia agar medium plates (as many as five presumptive positive and five presumptive negative colonies per sample type, per agar medium) with a sterile wooden applicator stick. These plates were then grown overnight anaerobically in a 37 °C incubator. On the following day, individual colonies from these plates were then inoculated onto a sterile cotton pad with a sterile wooden applicator stick and a 0.1 mL aliquot of Acid Phosphatase Reagent was then pipetted onto the colony. If the mixture became a purple color after about a minute, it was scored confirmed positive by the acid AP test. If it did not become purple, it was scored confirmed negative by the AP test.

#### 2.5.2. Stormy Fermentation Confirmation 

During the latter sampling periods of the investigation, isolates obtained from the test agar media were further subjected to a secondary confirmation test for stormy fermentation in iron-milk medium tubes [14]. After the AP tests, a second colony from each of the Columbia agar medium plates was inoculated into a glass tube of ~9 mL of iron-milk medium that was clearly marked to correspond with the water sample and agar medium from which it originally came as well as its result from the acid-phosphatase test. The inoculated tubes were then incubated for 24 h in a 44 °C incubator and checked for a positive stormy fermentation reaction in the medium. *C. perfringens* and other sulfite-producing clostridia are positive for stormy fermentation. Results were recorded accordingly.

### 2.6. C. perfringens Data Analysis Procedures

The data collected on *C. perfringens* concentrations were analyzed initially using the GraphPad Instat (version 3.1) statistical package from GraphPad Software (version 6.0). All data were analyzed using nonparametric tests, including the Friedman one-way analysis of variance test and the Dunn multiple comparison post-test because the data were not normally distributed. These tests were performed on direct colony count results obtained through membrane filtration on the three previously described selective media. The analysis was conducted for both unpasteurized and pasteurized samples (vegetative cells and spores and only spores, respectively) from diluted raw sewage, partially treated sewage (before sand filtration) and tertiary treated reclaimed water. To compare the concentrations of *C. perfringens* detected by TSC and CPCS agars in raw sewage samples, a Wilcoxon matched-pairs signed-ranks test was used. An alpha value of 0.05 *(p* < 0.05), was used to establish statistical significance (Table 1, Supplemental Table S2). 

For analysis of the confirmation test results, data were analyzed in Microsoft Excel and Stata 14 (StataCorp 2015). In Excel, the data were analyzed to determine the extent to which each *C. perfringens* confirmation test result agreed with what the isolate was considered presumptively (either *C. perfringens* positive or negative) when originally observed and the colony isolated from its respective agar medium. Microsoft Excel was then used to calculate sensitivities, specificities, positive predictive values (PPV), and negative predictive values (NPV). The confirmation tests were also compared against each other using McNemar’s test in Stata14 (StataCorp 2015) to determine if the results of the tests were significantly different from each other.

## 3. Results

The geometric mean of total *C. perfringens* concentrations in secondary treated effluent as detected by the TSC, CPCS, and m-CP media were 263 colony forming units (CFU)/100 mL (Standard Deviation [SD]: 147), 673 CFU/100 mL (SD: 670), and 167 CFU/100 mL (SD: 263), respectively (Table 1). The median *C. perfringens* values were 285 CFU/100 mL, 636 CFU/100 mL and 185 CFU/100 mL for each respective agar. For the same secondary treated samples, the ranges of *C. perfringens* concentrations on TSC agar were from 6 to 615 CFU/100 mL, on CPCS agar from 161 to 2285 CFU/100 mL, and on m-CP agar from 11 to 855 CFU/100 mL. TSC had the smallest range for detection of total *C. perfringens* cells in secondary treated wastewater. CPCS detected the largest range of *C. perfringens* compared with the other agars, mostly due to the high and low concentration outlier observations.

In the pasteurized untreated sewage samples, the average and median concentrations of spores on TSC, CPCS, and m-CP agar media were as follows: 3.27 × 10^4^ (SD: 3.19 × 10^4^) and 4.3 × 10^4^ CFU/100 mL on TSC, 3.87 × 10^4^ (SD: 7.64 × 10^4^) and 4.17 × 10^4^ CFU/100 mL on CPCS, and 4.73 × 10^3^ (SD: 1.40 × 10^2^) and 3.3 × 10^3^ CFU/100 mL on m-CP, respectively. *C. perfringens* spore concentrations in these samples of pasteurized untreated sewage ranged from 8.3 × 10^2^ to 1.3 × 10^5^ CFU/100 mL on TSC, 1.7 × 10^3^ to 3.2 × 10^5^ CFU/100 mL on CPCS, and 5.6 × 10^2^ to 1.8 × 10^4^ CFU/100 mL on m-CP, respectively.

For reclaimed water, no *C. perfringens* colonies were detected in the samples from the four treatment plants with combined chlorine and UV disinfection. In three samples from treatment plant C, with only UV disinfection of tertiary treated sewage, *C. perfringens* colonies were detected on all three agar media, with 40, 70, and 22 CFU/100 mL on TSC, 20, 40, and 46 CFU/100 mL on CPCS, and 3, 10, and 39 CFU/100 mL on m-CP, respectively.

To determine the log_10_ reductions in total *C. perfringens* cells and *C. perfringens* spores at each treatment plant, the average log_10_ concentrations of the tertiary treated reclaimed water were subtracted from the average log_10_ concentrations of the untreated sewage. All treatment plants’ tertiary treatment processes included dual disinfection with UV and chlorine, except for plant C which used single disinfection with UV radiation. The average log_10_ reductions in total *C. perfringens* cells for each of the treatment plants based on the CPCS agar, which had the highest enumeration levels of *C. perfringens*, were 4.60, 4.57, 4.02, 4.68, and 4.83 for treatment plants A, B, C, D, and E, respectively. Likewise, the average log_10_ reductions in *C. perfringens* spores for each of the treatment plants based on the CPCS agar, which had the highest enumeration levels of *C. perfringens*, were 4.61, 4.33, 4.31, 4.36, and 4.49 for treatment plants A, B, C, D, and E, respectively.

A Friedman nonparametric test was used to compare the concentrations of *C. perfringens* total cells and only spores as detected by the TSC, CPCS, and m-CP agars in secondary treated wastewater (Supplemental Table S2). This test indicated that statistically significant concentrations of *C. perfringens* total cells and spores were detected by the three agars. Following the Friedman test, a Dunn multiple comparison post-test was performed to compare each of the agar pairs individually (Table 2). In the secondary treated sewage sample, the CPCS agar resulted in a statistically significantly greater detection of total *C. perfringens* cells compared to the TSC and m-CP agar (Table 2; *p*-value < 0.001). Both the CPCS and TSC agars resulted in statistically significantly higher detection rates of *C. perfringens* spores than the m-CP agar (Table 2; *p*-value < 0.001). However, there was no statistically significant difference between the TSC and CPCS agars in detection of *C. perfringens* spores (Table 2; *p*-value > 0.05).

Compared across the first 11 raw sewage samples, both the TSC and CPCS agars resulted in significantly higher concentrations of spores and total cells of *C. perfringens* than the m-CP agar. Following the initial statistical analyses of these samples, the next eight untreated sewage samples were analyzed only with the CPCS and TSC agars and not the m-CP agar. Following the Dunn multiple comparison sample analyses, a Wilcoxon matched pairs signed-rank test was performed to compare the difference in *C. perfringens* spores and total cell concentrations in untreated sewage, detected by the TSC and CPCS agars. By these analyses, the CPCS agar detected a statistically significantly higher concentration of total *C. perfringens* cells in raw sewage than did TSC agar (*p* = 0.0015). However, there was no statistically significant difference between the two agars in *C. perfringens* spore concentration detection in raw sewage (*p* = 0.2101).

Presumptive positive and negative *C. perfringens* colony isolates for pre-sand filtered samples and untreated sewage samples were collected from each of the three test agar media for both pasteurized (*C. perfringens* spores) and unpasteurized (total *C. perfringens*) samples. Isolated colonies from each medium were subjected to confirmatory testing by AP production and SF in iron milk medium. In total, 533 presumptive isolates (275 presumptive positive colonies and 258 presumptive negative colonies) from pasteurized and unpasteurized secondary treated effluent and 303 presumptive isolates (171 presumptive positive colonies and 132 presumptive negative colonies) from pasteurized and unpasteurized untreated sewage were subjected to both confirmation tests (Supplemental Table S3). 

The results of the two *C. perfringens* confirmation tests indicated varying levels of agreement among the agar media. Additionally, the results of the tests for AP production and for SF in iron-milk medium differed among many of the presumptive isolates tested. Because of the differing results, specificities and sensitivities were generated based on each individual confirmation test and an agreement between the two confirmation tests. The sensitivities, specificities, positive predictive values (PPV) and negative predictive values (NPV) were calculated for each agar medium based on the results of the two confirmation tests for both pasteurized and unpasteurized raw sewage, secondary treated effluent, and total combined samples (Supplemental Tables S4 and S5). The overall sensitivities of TSC, CPCS, and m-CP agar media as determined by agreement of both confirmation tests on presumptive positive and presumptive negative *C. perfringens* isolates from both types of sewage samples were 0.81, 0.78, and 0.81 respectively. For the same agar media, their overall specificities as determined by agreement of both confirmation tests on *C. perfringens* isolates from both types of samples were 0.28, 0.81, and 0.97, respectively. The sensitivities of the TSC, CPCS, and m-CP agar media for pasteurized samples were 0.79, 0.81, and 0.63 while the specificities were 0.23, 0.65, and 0.98, respectively.

The overall PPV of TSC, CPCS, and m-CP agar media as determined by agreement of both confirmation tests on presumptive positive and presumptive negative *C. perfringens* isolates from both types of sewage samples were 0.53, 0.83, and 0.97, respectively. For the same agar media, their overall NPVs as determined by agreement of both confirmation tests on *C. perfringens* isolates from both types of samples were 0.59, 0.75, and 0.82, respectively. The PPVs of the TSC, CPCS, and m-CP agar media as determined by the same method for combined pasteurized samples were 0.53, 0.76, and 0.97 while the NPVs were 0.50, 0.72, and 0.74, respectively (Supplemental Table S5). Overall, PPVs and NPVs were least on TSC agar, compared to the other two agar media.

## 4. Discussion

The results from the statistical tests comparing the performance of the three agar media demonstrate that there are significant differences with respect to the ability to detect presumptive *C. perfringens* total cells and spores. The results of this study support previous findings that TSC is a superior agar to m-CP agar in its ability to detect and quantify *C. perfringens* in different types of water and wastewater samples [10,11,15]. The results of this study also demonstrate that CPCS agar can enumerate total *C. perfringens* cells at higher levels than the two other agar media tested in both types of wastewater samples analyzed, which has not been demonstrated in previous studies. This study found CPCS agar to produce higher detection of *C. perfringens* spores than m-CP agar, similar to other evaluations of m-CP which found that m-CP resulted in lower colony counts [7]. *C. perfringens* spore detection by CPCS agar was also comparable to TSC agar which is similar to the findings of previous work comparing CPCS agar and TSC agar with a fluorogenic substrate [11]. In a comparison of m-CP and TSC in surface waters, it was found that higher recoveries were obtained using TSC agar, a result that agreed with our findings in reclaimed and waste waters [6].

While TSC and CPCS agar may not have differed significantly in their ability to detect *C. perfringens* spores, the results of the two confirmation tests for presumptive *C. perfringens* colonies suggest that there may be differences in the reliability of these agar media to accurately detect biochemically and phenotypically confirmed *C. perfringens* colonies and true negative colonies. The finding of somewhat reduced sensitivity in *C. perfringens* detection for the TSC agar has been noted previously [10,16]. Other work has also noted that compared with m-CP agar, TSC agar does produces fewer false positives but also a higher number of false negatives [5]. The high sensitivity of m-CP for *C. perfringens* has also been previously observed, but the lower specificity of m-CP for *C. perfringens* detection is in contrast to some previous findings [3]. Overall, the values close to 80% specificity found for each of these agar media agrees with previous work that has found varying degrees of specificity among these agar media [10,16]. However, this study is the first to recognize that these similar specificities (0.81 for unpasteurized samples as determined by dual confirmation and 0.65 for pasteurized samples as determined by dual confirmation) are also found for *C. perfringens* detection by CPCS agar in raw and treated wastewater, as they have been reported previously for other waters.

Although presumptive *C. perfringens* colony isolates were tested by biochemical and phenotypic confirmation, there were moderate levels of disagreement between the two confirmation tests, with 143 cases of discordant results between the confirmation tests of the 836 (17.1%) total isolates subjected to confirmation testing (Supplemental Table S3). Overall, there was a significant difference between the results of the two confirmation tests according the McNemar’s tests (*p*-value = 0.024). While molecular confirmation of these isolates was not conducted for this research, previous studies have found that the phenotypic or biochemical confirmation tests are not always accurate, although the test for acid-phosphatase production has provided higher levels of measured sensitivity than the test for stormy fermentation [17,18,19,20]. These findings may explain why the two confirmation tests had varying levels of disagreement at times. To better assess the accuracy of these confirmation tests and the true sensitivities and specificities of the test agar media, confirmation of the presumptive isolates should be conducted by additional biochemical testing and through molecular analyses, such as molecular characterization and identification by nucleic acid analysis and by protein targeted mass spectrometry, such as MALDI-TOF MS using identity comparison to robust databases.

Although the findings of this research as stated above are supported by the analysis of many different wastewater samples, there are several limitations to this work. For some agar media, an estimated concentration value was used to represent the result on an agar medium plate that had either no growth or too numerous to count. These assigned values of lower (no detection) and upper (too numerous to count [TNTC]) censored results may have overrepresented or underrepresented the ability of the agar medium to detect and quantify *C. perfringens* in each respective wastewater sample. The use of a molecular based confirmation analysis of presumptive *C. perfringens* isolates, such as MALDT-TOF MS or PCR followed by nucleotide sequencing, would have provided a more definitive basis for confirmation of true positives.

Future research should consider molecular confirmation to compare to positive confirmation of presumptive isolates by the two confirmation tests used, acid phosphatase and stormy fermentation. Additionally, the TSC agar with the fluorogenic substrate that provides a rapid phenotypic phosphatase confirmation test should be evaluated in untreated and treated sewage samples to compare its performance and determine whether it could be a feasible alternative to the CPCS medium and whether the specificity on this medium would be as low as its TSC counterpart. More work should also be performed to compare the efficacy of these direct count methods for enumeration of *C. perfringens* with quantal most-probable-number methods such as stormy fermentation in iron-milk media in the context of wastewater and treated wastewater, as such studies have not previously been conducted.

## 5. Conclusions

Though there are many factors to consider when deciding which agar medium is the best for identifying and quantifying *C. perfringens* in wastewater and treated wastewater, the results of this study suggest that the CPCS agar is the best option based on its ease of use and ability to detect and differentiate colonies. This ease of use is primarily characterized by the ability to identify the distinctive green colonies. However, the CPCS agar is also the only one that requires boiling for preparation, whereas the TSC and m-CP media both require autoclaving before adding supplements. This difference did make the CPCS media qualitatively easier to prepare, given that it was less time consuming. Furthermore, the relatively high confirmation rates of presumptive positive isolates as determined by the tests for acid-phosphatase production and stormy fermentation in iron-milk medium support the reliable performance of this agar. Despite this, the TSC agar may be a suitable alternative if the interested lab is looking only at presence of spores in their water samples. This may frequently be the case as the spores are the intended surrogate indicator for the presence of protozoan parasites in water and highly treated wastewater. Because no statistically significant difference was found between the ability of TSC and CPCS agars to detect *C. perfringens* spores, it is likely that these two media can be used interchangeably for this purpose. This could be useful for resource limited labs that may not be able to afford the more expensive CPCS medium. However, the extremely low specificity determined for the TSC agar and the occasional occurrence of high counts of presumptive negative colonies suggest that, when it is possible, the CPCS agar medium is still preferred for use in obtaining the most accurate level of *C. perfringens* spore concentrations.

## Figures and Tables

**Table 1:** Central tendency statistics and ranges for concentrations of presumptive *C. perfringens* detected by TSC, CPCS, and m-CP agars in pasteurized and unpasteurized samples of untreated sewage and secondary treated effluent.

Central Tendency Statistic and Range	Unpasteurized Untreated Sewage (*n* = 19)			Pasteurized Untreated Sewage (*n* = 19)		
	TSC	CPCS	m-CP	TSC	CPCS	m-CP
Geometric Mean (CFU/100 mL)	5.36 × 10^4^	7.73 × 10^4^	1.54 × 10^4^	3.27 × 10^4^	3.87 × 10^4^	4.73 × 10^3^
Median (CFU/100 mL)	5.33 × 10^4^	6.33 × 10^4^	1.67 × 10^4^	4.33 × 10^4^	4.17 × 10^4^	3.33 × 10^3^
Minimum Value (CFU/100 mL)	1.20 × 10^4^	3.50 × 10^4^	5.56 × 10^3^	8.33 × 10^2^	1.67 × 10^3^	5.56 × 10^2^
Maximum Value (CFU/100 mL)	1.47 × 10^5^	3.58 × 10^5^	5.97 × 10^4^	1.27 × 10^5^	3.17 × 10^5^	1.83 × 10^4^
	Unpasteurized Secondary Treated Effluent (*n* = 25)			Pasteurized Secondary Treated Effluent (*n* = 25)		
	TSC	CPCS	m-CP	TSC	CPCS	m-CP
Geometric Mean (CFU/100 mL)	263	673	167	176	332	16
Median (CFU/100 mL)	285	636	185	233	267	21
Minimum Value (CFU/100 mL)	6	161	11	2	42	1
Maximum Value (CFU/100 mL)	615	2285	855	452	1535	206

**Table 2:** Results of the Dunn multiple comparison post-test comparing the matched concentrations of total *C. perfringens* and spores in secondary treated sewage effluent as detected by TSC, CPCS, and m-CP agars.

Agar 1	Agar 2	Sample Type	Dunn Multiple Comparison *p*-Value (*p*-Value < 0.05 is Significant) (Raw Data)	Rank Sum Difference (Raw Data)
**TSC**	**CPCS**	2^0^ treated sewage	<0.001	−30
**TSC**	**m-CP**	2^0^ treated sewage	>0.05 *	9
**CPCS**	**m-CP**	2^0^ treated sewage	<0.001	39
**TSC**	**CPCS**	2^0^ treated sewage Δ	>0.05 *	−12
**TSC**	**m-CP**	2^0^ treated sewage Δ	<0.001	30
**CPCS**	**m-CP**	2^0^ treated sewage Δ	<0.001	42

Δ-indicates pasteurization of sample at 65°C for 15 min (or *C. perfringens* spores sample). * indicates statistical significance.

## Data Availability

Data are available from the corresponding author upon reasonable request.

## Supplementary Materials

The following supporting information can be downloaded at: https://www.mdpi.com/article/10.3390/pathogens13070526/s1, Table S1: Number and type of samples analyzed by treatment plant (samples collected May–July 2013), Table S2: Results of Friedman test comparing the matched concentrations of total C. perfringens and spores in secondary treated sewage effluent as detected by TSC, CCP, and m-CP agars, Table S3: Summary of the number of presumptive isolates tested for acid phosphatase production and stormy fermentation in iron milk from each agar type and sample type, Table S4: Sensitivities and specificities of each agar as determined by agreement of presumptive isolates with phenotypic confirmation testing for acid phosphatase production or stormy fermentation in untreated sewage and secondary treated effluent samples, Table S5: Positive Predictive Values (PPV) and Negative Predictive Values (NPV) of each agar as determined by agreement of presumptive isolates with phenotypic confirmation testing for acid phosphatase production or stormy fermentation in untreated sewage and secondary treated effluent samples.
