# Supplementary material for: Evaluation of Three Different Selective Media for Enumeration of Clostridium perfringens in Untreated and Treated Wastewater
Source: Pathogens. 2024 Jun 21;13(7):526. doi: 10.3390/pathogens13070526 (PMC11279483; doi:10.3390/pathogens13070526)
Supplement: Supplementary file 1 [file pathogens-13-00526-s001.zip › pathogens-3015240-supplementary.pdf]

Supplementary Table S1. Number and type of samples analyzed by treatment plant.

Treatment Plant	Untreated Raw Sewage (Number of Samples)	Secondary Treated Effluent before Sand Filtration (Number of Samples)	Tertiary Treated Reclaimed Water (Number of Samples)	TOTALS
A	4	8	6	18
B	3	5	5	13
C	4	4	5	13
D	4	5	5	14
E	4	3	4	12

Supplementary Table S2. Results of Friedman test comparing the matched concentrations of total *C. perfringens* and spores in secondary treated sewage effluent as detected by TSC, CCP, and m-CP agars.

**Sample type**	**Friedman test p-value (p-value < 0.05 is significant)**
2^0 treated sewage, no treatment (total *C. perfringens*)	<0.0001
2^0 treated sewage, pasteurized (*C. perfringens* spores)	<0.0001

Supplementary Table S3. Summary of the number of presumptive isolates tested for acid phosphatase production and stormy fermentation in iron milk from each agar type and sample type.

**Sample**	**Number of Presumptive Isolates Tested**								
	Agar	Negative	Positive	Agar	Negative	Positive	Agar	Negative	Positive
Secondary Treated Effluent	TSC	50	50	m-CP	39	47	CPCS	46	50
	TSCΔ	49	50	m-CPΔ	35	28	CPCPΔ	39	50
Raw Sewage	TSC	30	30	m-CP	22	30	CPCS	21	30
	TSCΔ	24	30	m-CPΔ	17	21	CPCSΔ	18	30

(Δ-designates that the samples were pasteurized to give detection of *C. perfringens* spores only)

Supplementary Table S4. Sensitivities and specificities of each agar as determined by agreement of presumptive isolates with phenotypic confirmation testing for acid phosphatase production or stormy fermentation in untreated sewage and secondary treated effluent samples.

		**Agar Type and Sample Treatment**					
		TSC	CPCS	m-CP	TSC Δ	CPCSΔ	m-CPΔ
**Acid Phosphatase**							
Secondary Treated Effluent	Sensitivity	0.86	0.86	0.87	0.86	0.90	0.82
	Specificity	0.34	0.91	1.00	0.32	0.79	1.00
Raw Sewage	Sensitivity	0.87	0.90	0.87	0.80	0.80	0.71
	Specificity	0.37	0.77	0.86	0.46	0.89	1.00
Combined Secondary Treated Effluent and Sewage	Sensitivity	0.86	0.88	0.87	0.84	0.86	0.78
	Specificity	0.38	0.94	0.99	0.36	0.82	1.00
**Stormy Fermentation**							
Secondary Treated Effluent	Sensitivity	0.92	0.92	0.87	0.90	0.96	0.75
	Specificity	0.32	0.81	0.98	0.38	0.74	1.00
Raw Sewage	Sensitivity	0.93	0.77	0.93	0.87	0.83	0.86
	Specificity	0.37	0.86	0.95	0.29	0.78	1.00
Combined Secondary Treated Effluent and Sewage	Sensitivity	0.93	0.86	0.90	0.89	0.91	0.80
	Specificity	0.34	0.82	0.97	0.35	0.75	1.00
**Acid Phosphatase & Stormy Fermentation**							
Secondary Treated Effluent	Sensitivity	0.82	0.82	0.81	0.84	0.90	0.68
	Specificity	0.26	0.79	0.98	0.24	0.64	1.00
Raw Sewage	Sensitivity	0.80	0.70	0.80	0.70	0.67	0.57
	Specificity	0.30	0.86	0.95	0.21	0.67	0.94
Combined Secondary Treated Effluent and Sewage	Sensitivity	0.81	0.78	0.81	0.79	0.81	0.63
	Specificity	0.28	0.81	0.97	0.23	0.65	0.98

(Δ-designates that the samples were pasteurized to give detection of *C. perfringens* spores only)

Supplementary Table S5. Positive Predictive Values (PPV) and Negative Predictive Values (NPV) of each agar as determined by agreement of presumptive isolates with phenotypic confirmation testing for acid phosphatase production or stormy fermentation in untreated sewage and secondary treated effluent samples.

		**Agar Type and Sample Treatment**					
		TSC	CCP	m-CP	TSC Δ	CCPΔ	m-CPΔ
**Acid Phosphatase**							
Secondary Treated Effluent	PPV	0.55	0.91	1.00	0.55	0.83	1.00
	NPV	0.65	0.84	0.94	0.63	0.84	0.88
Raw Sewage	PPV	0.59	1.00	0.96	0.60	0.87	0.92
	NPV	0.69	0.86	0.84	0.28	0.67	0.73
Combined Secondary Treated Effluent and Sewage	PPV	0.57	0.94	0.98	0.57	0.84	0.97
	NPV	0.67	0.85	0.87	0.57	0.78	0.82
**Stormy Fermentation**							
Secondary Treated Effluent	PPV	0.55	0.82	0.97	0.58	0.80	1.00
	NPV	0.76	0.90	0.89	0.63	0.93	0.83
Raw Sewage	PPV	0.56	0.88	0.96	0.55	0.83	1.00
	NPV	0.82	0.72	0.91	0.56	0.75	0.80
Combined Secondary Treated Effluent and Sewage	PPV	0.55	0.84	0.97	0.62	0.81	1.00
	NPV	0.79	0.83	0.89	0.61	0.87	0.82
**Acid Phosphatase & Stormy Fermentation**							
Secondary Treated Effluent	PPV	0.53	0.80	0.97	0.53	0.75	1.00
	NPV	0.59	0.80	0.84	0.60	0.84	0.80
Raw Sewage	PPV	0.53	0.88	0.96	0.53	0.77	0.92
	NPV	0.60	0.67	0.78	0.36	0.55	0.64
Combined Secondary Treated Effluent and Sewage	PPV	0.53	0.83	0.97	0.53	0.76	0.97
	NPV	0.59	0.75	0.82	0.50	0.72	0.74

(Δ-designates that the sample plates on this agar was pasteurized)
